# An E-Learning Module to Improve Nongenetic Health Professionals’ Assessment of Colorectal Cancer Genetic Risk: Feasibility Study

**DOI:** 10.2196/mededu.7173

**Published:** 2017-12-18

**Authors:** Kirsten Freya Lea Douma, Cora M Aalfs, Evelien Dekker, Pieter J Tanis, Ellen M Smets

**Affiliations:** ^1^ Department of Medical Psychology Academic Medical Center University of Amsterdam Amsterdam Netherlands; ^2^ Department of Clinical Genetics Academic Medical Center University of Amsterdam Amsterdam Netherlands; ^3^ Department of Gastroenterology and Hepatology Academic Medical Center University of Amsterdam Amsterdam Netherlands; ^4^ Department of Surgery Academic Medical Center University of Amsterdam Amsterdam Netherlands

**Keywords:** colorectal neoplasms, colorectal, neoplasms, hereditary nonpolyposis, adenomatous polyposis coli, genetic testing, gastroenterology, health communication, feasibility studies, education, professional

## Abstract

**Background:**

Nongenetic health providers may lack the relevant knowledge, experience, and communication skills to adequately detect familial colorectal cancer (CRC), despite a positive attitude toward the assessment of history of cancer in a family. Specific training may enable them to more optimally refer patients to genetic counseling.

**Objective:**

The aim of this study was to develop an e-learning module for gastroenterologists and surgeons (in training) aimed at improving attitudes, knowledge, and comprehension of communication skills, and to assess the feasibility of the e-learning module for continued medical education of these specialists.

**Methods:**

A focus group helped to inform the development of a training framework. The e-learning module was then developed, followed by a feasibility test among a group of surgeons-in-training (3rd- and 4th-year residents) and then among gastroenterologists, using pre- and posttest questionnaires.

**Results:**

A total of 124 surgeons-in-training and 14 gastroenterologists participated. The e-learning was positively received (7.5 on a scale of 1 to 10). Between pre- and posttest, attitude increased significantly on 6 out of the 10 items. Mean test score showed that knowledge and comprehension of communication skills improved significantly from 49% to 72% correct at pretest to 67% to 87% correct at posttest.

**Conclusions:**

This study shows the feasibility of a problem-based e-learning module to help surgeons-in-training and gastroenterologists in recognizing a hereditary predisposition in patients with CRC. The e-learning led to improvements in attitude toward the assessment of cancer family history, knowledge on criteria for referral to genetic counseling for CRC, and comprehension of communication skills.

## Introduction

Colorectal cancer (CRC) is one of the most common forms of cancer worldwide. Mortality can be reduced if individuals at risk are detected and treated early [1]. Patients with a familial or hereditary risk (eg, Lynch syndrome and familial adenomatous polyposis) comprise about 10% to 30% of all patients with CRC [2,3]. Recognition of hereditary CRC syndromes helps to identify high-risk patients, provide them with appropriate surveillance, and offer surgical options. Despite the relatively high frequency of familial and hereditary CRC syndromes and the proven benefit of screening high-risk individuals, referral for genetic counseling appears to be suboptimal, leading to under-diagnosis of hereditary CRC [4].

Adequate referral may be hampered by the clinician’s lack of *knowledge* on who and when to refer, and on lack of *experience and training* [4,5]. Indeed, some health professionals (such as gastroenterologists and surgeons) are not specifically trained in genetics and may lack the experience to adequately discuss genetic issues with their patients [6]. In the Netherlands, gastroenterologists and surgeons (rather than primary care physicians) function as gatekeepers for patients with CRC, identifying most patients at risk and providing them with initial information on heredity and genetic testing [6]. Hereafter, we refer to these health professionals as nongenetic health professionals.

It is reported that, in 80% of consultations, the oncologists, surgeons, and gastroenterologists have discussed the family history [6-8]. This suggests a positive *attitude* toward the assessment of a family history. However, 1 study showed that the quality of these discussions on family history of cancer may be inadequate (58%) [6]. The limited quality was mainly attributed to inadequate *communication skills*, for example, the clinicians asked vague, incomplete, overly general, and steering questions, or multiple questions at one time. In addition, when clinicians addressed patients’ family history, an increased risk for CRC was only discussed in 57% of those patients for whom such a discussion was warranted [7]. As a consequence, patients with an indication for genetic counseling may have been missed.

These studies suggest that nongenetic health providers may lack relevant knowledge, experience, and communication skills to adequately detect familial CRC, despite a possibly positive attitude toward family history assessment [4-7]. Dedicated training may enable them to adequately refer patients for genetic counseling [6]. Thus, training for nongenetic health care professionals should not only increase factual knowledge but also improve knowledge on effective communication with regard to genetics [9,10].

For gastroenterologists and surgeons (residents as well as specialists), (continued) medical education is traditionally organized through conferences, courses, workshops, and educational meetings. However, as specialists (in training) have limited time and the skill of discussing hereditary risks is a relatively small part of daily care, e-learning may be of practical value.

E-learning can be defined as a training, education, or instruction that occurs on a digital medium, such as a computer or mobile device [11]. The advantages of e-learning are that it is flexible, inexpensive, easy to adapt to individual needs and newest insights, and can be completed at any self-chosen time and location; moreover, e-learning can be adapted to the newest technological advances [11-13]. However, a disadvantage is that the translation of the skills learned into clinical practice may be less obvious. Only a few studies have investigated the effect of e-learning on communication skills. For example, McCarthy et al showed an improvement of skills in knowing when and how to complete incident forms and disclosing errors [14]. Daetwyler et al showed that their e-learning module improved the skill of breaking “bad news” in a setting with a simulated patient [15]. Another study that aimed to improve clinicians’ behavior during genetic testing for ovarian cancer showed that a change in knowledge through Web-based learning can drive behavior change [16].

This study addresses the feasibility of e-learning aimed at the improvement of attitude, knowledge, and communication skills in health professionals. Specifically, the aims were as follows: (1) to develop an e-learning module for gastroenterologists and surgeons (in training) aimed at improving *attitudes* toward assessment of a cancer family history, *knowledge* on hereditary CRC and criteria for referral to genetic counseling, and *comprehension of communication skills* (ie, insight into the need to assess a cancer family history in a structured, nonsteering way); and (2) assess the feasibility of this e-learning module for continued medical education of these specialists.

This test of feasibility includes the perception of gastroenterologists and surgeons on the timing, time constraints, technical problems, fulfillment of expectations, clinical usability, and usefulness, as well as the design and technical usability of the e-learning module.

## Methods

This study consisted of development of the e-learning module and measurement of the feasibility of the e-learning module to allow improvements (if required).

### Stage 1: Development

#### Focus Group

First, as the target group is difficult to recruit, an online asynchronous focus group was organized. The purpose of this focus group was to investigate the attitudes of gastroenterologists and surgeons-in-training toward collecting a cancer family history and discussing genetic testing, and their need for an e-learning module to connect to their preferences and needs. Using purposeful sampling, the aim was to approach 6-12 gastroenterologists and surgeons-in-training of varying gender and experience.

The focus group discussion addressed the following: oncogenetic knowledge, attitude, perceived communication skills in and competencies and barriers toward collecting a cancer family history and discussing genetic testing, knowledge on information sources about hereditary CRC, organizational aspects associated with either investigating or not investigating a cancer family history and discussing genetic testing, available tools for discussing a cancer family history, and educational preferences (ie, what educational elements should e-learning entail).

The focus group discussion was carried out by means of the free Web tool FocusGroupIt, LLC (Matt Foley, Rochester, NY, USA) [17]. Focus group participants were invited to join the discussion at several times during a 10-day period. They were instructed to answer the moderator’s (KD) open questions in a predefined order and were encouraged to react to each other’s answers. The moderator asked questions to further stimulate the discussion. An inductive approach, based on the questions addressed, allows to summarize what the health professionals said. The summary of the transcript of the discussion was shared with the participants to enable their feedback.

#### Framework Development

On the basis of the input of the focus group, a framework for the e-learning module was developed by the study coordinator (KD) in close collaboration with a clinical geneticist (CA), surgeon (PT), gastroenterologist (ED), medical psychologist (ES), educational expert (EtP), and an e-learning developer (PD). The framework comprised descriptions of the content (*what?*), learning goals (*why?*), and method (*how?*) for each step of the e-learning module. The learning goals were formulated using the taxonomy of Bloom [18], which consists of six levels of learning in the cognitive domain (evaluation, synthesis, analysis, application, comprehension, and knowledge). On the basis of this framework, a script was written.

#### E-Learning Module Development

In the next step, the e-learning module itself was developed. In this phase, choices and decisions regarding the medical content and the configuration of the e-learning module were made. Technical decisions were made regarding navigation (learner or program-controlled), use of multimedia (verbal, visual, or audio), use of game elements, and which software to use. After the development of the e-learning module, 8 professionals (including medical experts such as a gastroenterologist, surgeon, and clinical geneticist) and researchers specialized in medical communication tested the first version of the e-learning module. They critically commented on the content (depending on their expertise), the language used, and the ease of use of the module.

### Stage 2: Feasibility

#### Study Participants

Feasibility testing of the module was first performed among a group of surgeons-in-training (3rd- and 4th-year residents; hereafter called surgeons) and then among gastroenterologists. For feasibility studies, a sample size of at least 55 participants has been recommended [19].

#### Procedure

Separate procedures were followed for each of the two groups. In spring 2016, the surgeons participated in an obligatory national training day on oncology. In addition, 1 week before the training, they were invited via an email from the organizers of the oncology training, strongly recommending their participation in the e-learning module. Surgeons were asked to fill in a brief online questionnaire both before (T0) and directly after (T1) the e-learning module. The email contained a link to the first questionnaire. At the end of the questionnaire, a link to a website with the e-learning module was provided. Participants were able to access the e-learning module at a time and place of their own convenience but were asked to complete it before their national training day. The link to the second questionnaire was provided at the end of the e-learning module.

On the basis of the input from the surgeons, adaptations could be made (if required) to the e-learning module before submitting it to the gastroenterologists. Using a list of all gastroenterologists registered in the Netherlands, this group was directly approached by the principal researcher via email. The email contained a link to the first questionnaire. At the end of the questionnaire, a link to a website with the e-learning module was provided. Gastroenterologists were also able to access the e-learning module at a time and place of their own convenience but within a time limit of 2 weeks after the invitation. The link to the second questionnaire was provided at the end of the e-learning module. Participants could not enter the second questionnaire without having completed the prequestionnaire and the e-learning module.

All surgeons and gastroenterologists received a gift voucher of 30 euro for their participation to compensate for their time, owing to their busy schedules.

#### Measures

Table 1 shows the items included in the pre- and postquestionnaire. The prequestionnaire assessed the following: personal characteristics (age, gender, year of graduation, and experience with the patient population), attitude toward cancer family history assessment (Continuing Professional Development Reaction Questionnaire), and expectations regarding the e-learning module. A self-developed knowledge test on hereditary CRC and communication skills, consisting of a pre- and posttest, was part of the e-learning module.

In the postquestionnaire, participants were invited to evaluate the e-learning module on the timing, time constraints, technical problems, fulfillment of expectations, clinical usability and usefulness, design and technical usability, and attitude. Questions based on the study of other authors were translated by the study coordinator (KD) and checked for content validity by a clinical geneticist (CA), surgeon (PT), gastroenterologist (ED), and medical psychologist (ES). The questions regarding relevant knowledge were also checked for content validity by these experts. Participants were encouraged, but not obliged, to fill in the questionnaires directly before and after completing the e-learning module. Therefore, some time may have elapsed between the completion of the e-learning and the pre- and postquestionnaire.

#### Data Analyses

Descriptive statistics were used to analyze most aspects of feasibility. To determine a change in *attitude* and *knowledge* between T0 and T1 either a paired *t* test or Wilcoxon signed-rank test was used, depending on the distribution of the data. Data were analyzed using IBM SPSS Statistics 23 (IBM Corp).

**Table 1:** Measures included in the pre- and postquestionnaire.

Category		Number of items	Reference	Description of questions or response scale (if applicable)	Time point	
					Pre	Post
General characteristics		5	Self-developed	Age, gender, year of completion as physician, experience with patients with CRC^a^ (5-point scale: very much to none), and estimation of number of patients with CRC seen in last 3 months	X^b^	
Attitude, beliefs, and intentions		10	Based on the CPD^c^ Reaction Questionnaire [20]	Attitude, beliefs, and intentions toward collecting a cancer family history (different response scales depending on the item; see Table 4 for the items)	X	X
**Knowledge**						
	Tested	8	Self-developed	Eight questions on knowledge about hereditary CRC and assessment of cancer family history		X
	Self-evaluation	2	Based on Robinson et al 2015 [16]	Did your knowledge on hereditary CRC and investigating a cancer family history increase? (7-point scale: strongly disagree to strongly agree)		X
General evaluation of e-learning		4	Self-developed	Give a grade: 1 (low)-10 (high) Would you advise others to follow the e-learning module (yes or no or maybe)? Would you be willing to pay for such an e-learning module (none, 0-15, 15-30, or more than 30 euro)? Would you be interested in other modules (yes or no or maybe)?		X
Timing		1	Self-developed	Did the e-learning come at the right point in time during the educational track (only applicable for surgeons; 3-point scale: too early to too late)		X
Time constraints		2	Self-developed	How did you evaluate the length of the e-learning? (5-point scale: much too long to much too short) How long did it take you to complete the e-learning module? (0-15, 15-30, 30-45, 45-60, or more than 60 min)		X
Technical problems		4	Self-developed	On what device did you follow the e-learning? How many turns did you take to complete it? Did you encounter technical problems, and if so, what type of problems? (3 multiple choice and 1 open questions)		X
Design and technical usability		10	Based on Jacobs et al (personal communication, Ellen Smets, December 2016)	Did you think the e-learning was well-developed, user-friendly, nice, readable, and usable? Did you think the e-learning used understandable language, understandable instructions, clear instructions, useful instructions, and complete instructions? (5-point scale: not at all to very much)		X
**Clinical usability and content**						X
	Expectations	3	Based on te Pas et al [21]	See Table 6 for the items. (7-point scale: strongly disagree to strongly agree)	X	X
	Cases	4	Self-developed	Did you find the case examples used clear, helpful, complete and realistic? (7-point scale: not at all to very much)		X
	Content of e-learning	3	Self-developed	Which two components of the e-learning did you find most and less useful? Did you miss anything, and if yes, what did you miss? (2 multiple choice and 1 open questions)		X

^a^CRC: colorectal cancer.

^b^X means that the questionnaire was used at that time point.

^c^CPD: Continuing Professional Development.

## Results

### Stage 1: Development

#### Focus Group

A total of 2 gastroenterologists-in-training (1 male and 1 female; 1 in the final year of training and 1 at the start of training) and 5 surgeons-in-training (3 females and 2 males; 1 at the start of training, 2 in the middle of their training, and 2 in the final year of their training) participated in the online focus group. In addition, 1 gastroenterologist-in-training (male, in the middle of his training) was interviewed individually.

The participants had a positive attitude toward *collecting a family history*; however, not all of them had actual experience. They acknowledged the added value of a cancer family history assessment, such as investigating a differential diagnosis, evaluating the need to refer for genetic counseling, and identifying the potential risk for family members. One of their main barriers in the current practice was lack of time; participants worried that discussing cancer family history was potentially time-consuming because of the possible emotional reactions of patients. Another reported barrier was lack of oncogenetic knowledge; participants suggested that patient checklists or physician training may help overcome this barrier.

A total of 5 participants had experience in *discussing genetic testing* with patients, and all had perceived this as important. Lack of time, unclear procedures, lack of knowledge on guidelines, and referral criteria were reported as the most important barriers in discussing genetic testing with patients. To overcome these barriers, the use of a clear protocol, feedback of the clinical geneticist, education, and a checklist were suggested.

When prompted, participants indicated wanting to improve the following communication skills: asking concrete open questions, following through with questions, signaling cues, and clearly formulating and structuring questions about cancer in the family. However, most participants thought that an e-learning module should focus mainly on oncogenetic knowledge and not on communication skills. Participants reported that they were not thoroughly educated about genetics during their curriculum or training. An e-learning module on this topic would need to be short and problem- or case-based, and also discuss useful information sources.

#### E-Learning

On the basis of the input of the focus group, a framework for the e-learning module was developed; then, a prototype of the e-learning module was developed. Articulate storyline version 2.1 was used because of the experience with this software within our hospital. During the development process, the study coordinator (KD) and the e-learning developer (PD) continuously discussed the technical decisions to be made. Subsequently, this prototype was tested among 8 professionals; this led to only small changes, for example, correction of spelling or grammatical errors and some errors in medical content.

Table 2 shows the content of the e-learning, and Multimedia Appendix 1 shows some screenshots of an English translation of the Dutch e-learning module.

### Stage 2: Feasibility

#### Study Population

##### Surgical Residents

A total of 104 surgical residents were invited; however, 124 prequestionnaires (T0) were collected because several participants reentered the prequestionnaire after missing the link to the e-learning module. On the basis of a decision rule to distinguish those who had reentered the questionnaire from those who had not, we were able to exclude these questionnaires. The decision rule was formulated as follows: if gender, age, and Internet Protocol address were similar, answers on the remainder of the questionnaire differed on less than 6 variables, and year of graduation differed less than 2 years, then the last questionnaire filled in was removed. Unfortunately, because we were unable to identify all double entries, the final sample comprised 110 completed questionnaires at T0. At T1, 84 surgeons completed the questionnaire.

##### Gastroenterologists

Out of the 39 invited gastroenterologists, 14 participated (36% response rate) at T0 and 10 participated at T1. Reentry was not possible with this questionnaire. Pre- and postquestionnaires were mostly compared on a group level. However, for the pre- and posttest data, we paired the data for the 84 surgeons and the 10 gastroenterologists for which we had data at two points in time. Table 3 shows the sociodemographic characteristics of the study sample at T0.

#### Evaluation

Results of the 2 study groups are presented together, with the exception of differences between the versions of the e-learning module that could influence the results of the 2 groups, such as the game element.

On average, participants rated the e-learning module 7.5 (standard deviation [SD] 0.9) on a scale of 1 to 10. For the question “Would you recommend the e-learning to others like you?,” 75.5% (71/94) of the participants said yes, 16.1% maybe (15/94), and 7.5% (7/94) said no.

Out of the surgeons, 86% would be interested in e-learning modules about other hereditary cancers (eg, hereditary breast and ovarian cancer), 6% would not, and 8% “might be.” Out of the gastroenterologists, 44% would be interested in e-learning modules about other aspects of hereditary (eg, next generation sequencing or genomics), 22% would not, and 33% “might be.”

Out of all participants, 67% said they would not be willing to pay for the e-learning if they could get accreditation points for it, 26% would be willing to pay up to 15 euro, 7% would be willing to pay 15-30 euro, and 1% would be willing to pay more than 30 euros.

**Table 2:** Content of the e-learning module. For all questions, participants received standardized textual feedback based on their answers.

Topic	Explanation	Examples of questions within the topic
Entry test	The entry level of knowledge of the participant was tested with 8 multiple choice questions	Which advice is not relevant for adequately assessing a family history? Pick one.
		Response options were as follows: Ask about second-degree family members Ask for the age at which cancer in the family member was diagnosed Ask if there were metastases in cancers in the family
		
Long cases using comics with questions	Two clinical scenarios (one with a mistake in medical content, and one with a communication mistake) in the form of a comic with questions (see screenshots)	Do you have enough information to decide if this patient should be referred for genetic counseling?
		Response options were as follows: Yes, I have enough information. The patient should not be referred No, I do not have enough information Yes, I have enough information. The patient should be referred
		
		
Overview of helpful aids to assess cancer family history	Links to relevant information in apps, checklists, and questionnaires with 1 reflective question	Which method do you find most useful for clinical practice?
Four short cases	Case descriptions for which the participant needs to evaluate whether the patient needs to be referred for genetic counseling	A patient got bowel cancer at the age of 49 years and has a niece with endometrium cancer at the age of 60 years. Does this patient need to be referred for genetic counseling? yes or no
Communication examples	Examples of erroneous communication skills and reflective questions on how to improve questions (asking concrete open questions, following through with questions, signaling cues, and clearly formulating and structuring questions about cancer in the family) when investigating a cancer family history	*And something else. Nobody in your family has bowel cancer?*^a^ How could you rephrase this question?
Misunderstandings in two comics	Two clinical scenarios in which misunderstandings arise and multiple-choice questions about these misunderstandings	*I am planning to buy a house. Is it wise to get a DNA test done? I have heard that it can have consequences for your insurance and that you would not be able to buy a house.*
		What would be an appropriate response to the reaction of the patient? In most cases, genetic testing has no consequences for insurance. The clinical geneticist can discuss this with you and help you decide what is the most sensible thing to do You can better wait until you have bought your house. I will refer you to a clinical geneticist after you have done that DNA research has no consequences for your insurance. The clinical geneticist can tell you more about that
Misunderstanding in game or comics	Description of most common misunderstandings by patients about genetic testing, such as consequences for insurance, including the in-laws in the family history, etc. In the first version, participants had to click on rolling balls within a certain time frame to make the misunderstandings visible. This format was changed after the test among surgeons-in-training. In the second version, pictures of patients were shown with a text balloon reflecting their misunderstanding. An explanation of the misunderstanding and on how to deal with it was provided	Thinking balloon of patient: *“Cancer in the family she asks. Hmmm, what types of cancer do we have in my mother’s family?”* Advice for health professional: You can explicitly say to the patient that she needs to consider both sides of the family
Barriers word cloud	Participants could click on words in a word cloud presenting the most common barriers clinicians experience in discussing a cancer family history and genetic testing and how to overcome these	Word in the word cloud: Timing Explanation: At the time of diagnosis, there is a lot that needs to be discussed with a patient. However, because in some cases the genetic test result can influence the treatment, it is important to address the cancer family history early in the trajectory. Experience shows that when the topic is not addressed in the first consultation, it will not be discussed in follow-up consultations
More information (overview of helpful aids)	A downloadable overview of the most important information sources, for example, websites with guidelines and informative websites, for patients and health professionals	N/A^b^
End test	With the end test, the level of knowledge after following the e-learning was evaluated with the same 8 multiple choice questions as in the entry test	Which tumors are associated to Lynch? Answering options were as follows: Endometrium cancer Cervical cancer Biliary tract cancer Sebaceous gland carcinoma Hodgkin lymphoma
	

^a^Text in italics are expressions of hypothetical patients or doctors.

^b^N/A: not applicable.

**Table 3:** Characteristics of the respondents at T0.

Characteristics of the respondents (N=124)		Surgical residents (N=110)		Gastroenterologists (N=14)	
		Mean (range, SD^a^)	n (%)	Mean (range, SD)	n (%)
Age in years^b^		31.6 (28-37, 1.8)		36.2 (26-60, 9.5)	
**Gender**^c^					
	Male		65 (59.6)		6 (46.2)
	Female		44 (40.4)		7 (53.8)
Years since completing medical degree^d^		7 (3-11, 1.7)		11 (0-35, 9.5)	
**Experience with CRC**^e^					
	A lot or much		70 (63.6)		8 (57.1)
	Not much or little		39 (35.4)		5 (35.7)
	None		1 (0.9)		1 (7.1)
**Number of patients seen with CRC in the last 3 months**					
	0-19		36 (32.7)		12 (85.7)
	20-39		54 (49.1)		1 (7.1)
	40-59		9 (8.2)		
	60-79		4 (3.6)		1 (7.1)
	80-99		1 (0.9)		
	100 or more		6 (5.5)		

^a^SD: standard deviation.

^b^Missing values: 3

^c^Missing values: 2

^d^Missing values: 4

^e^CRC: colorectal cancer.

#### Attitude, Beliefs, and Intentions

Tables 4 and 5 show the attitude of participants toward asking for a cancer family history at T0 and T1.

Regarding participants’ beliefs about their capabilities that they would find it easy to ask for a cancer family history (*z*=−2.90, *P*=.004), participants were significantly less positive at T1 compared with T0. No differences were reported for their capability to ask for a cancer family history or their confidence in asking for a cancer family history.

Regarding participants’ perception of social influences that colleagues would ask for a cancer family history (*z*=−2.62, *P*=.009) and that persons who are important in their profession would ask for a cancer family history (*z*=−3.71, *P*=.000), participants were significantly more positive at T1 compared with T0. No significant differences were found with regard to whether the participants thought respected coworkers would ask for a cancer family history.

Concerning beliefs about consequences that asking for a cancer family history is useful from a medical point of view (*z*=−2.51, *P*=.012), participants were significantly more positive at T1 compared with T0.

Regarding moral norms, participants were significantly more positive that asking for a cancer family history is the right thing to do from a medical perspective (*z*=−2.73, *P*=.006) at T1 as compared with T0.

Concerning participants’ intention to ask for a cancer family history (*z*=−2.82, *P*=.005) and to plan to ask for a cancer family history (*z*=−6.72, *P*=.001), participants were significantly more positive at T1 compared with T0.

#### Knowledge on Hereditary Cancer and Comprehension of Communication Skills

For surgeons, the mean test score significantly improved from 49% correct (SD 21, range 0-100) on the pretest to 67% correct (SD 20, range 10-100) on the posttest (*t*_82_=−6.11, *P*<.01); 70% of the individual scores improved, 12% decreased, and 18% remained stable. For accredited e-learning modules, the posttest score should be at least 70% correct. Therefore, before inviting the gastroenterologists, we critically reviewed and slightly adapted the test and the content of the e-learning, so that they were better aligned.

For gastroenterologists, the mean test score significantly improved from 72% correct (SD 18, range 50-100) on the pretest to 87% correct (SD 11, range 70-100) on the posttest (*z*=−2.25, *P*=.02); 70% of the scores improved, 10% decreased, and 20% remained stable.

On average, participants self-rated their increase in knowledge (7-point Likert scale, strongly disagree to strongly agree) on hereditary CRC with a 3.7 (SD 2.0) and their comprehension on how to investigate a cancer family history with a 5.5 (SD 1.0).

**Table 4:** Attitude, beliefs, and intentions toward investigating a cancer family history.

Scale and item^a^		T0 (n=123) Mean (SD^b^)	T1 (n=94) Mean (SD)
**Beliefs about capabilities**			
	I have the ability to ask for a cancer family history (strongly disagree to strongly agree)	6.3 (0.7)	6.3 (0.6)
	I am confident that I could ask for a cancer family history (strongly disagree to strongly agree)	6.1 (1.0)	6.2 (0.7)
	For me, asking for a cancer family history would be (extremely difficult to extremely easy)	6.2 (0.8)	6.0 (0.7)
**Social influences**			
	To the best of my knowledge, the proportion of colleagues who will ask for a cancer family history would be (0%-20% or 20%-40% or 40%-60% or 60%-80% or 80%-100%)^c^	5.0 (1.4)	5.4 (1.1)
	Now think about a coworker who you respect as a professional. In your opinion, does he or she ask for a cancer family history (never to always)	5.7 (1.0)	5.8 (0.8)
	Most persons who are important for me in the profession would ask for a cancer family history (strongly disagree to strongly agree)	5.5 (1.0)	5.8 (0.8)
**Beliefs about consequences**			
	Overall, I think that asking for a cancer family history from a medical point of view is (useless to useful)	6.1 (0.9)	6.4 (0.6)
**Moral norm**			
	Asking for a cancer family history is the right thing to do from a medical perspective (strongly disagree to strongly agree)	6.1 (1.0)	6.4 (0.6)
**Intention**			
	I intend to ask for a cancer family history (strongly disagree to strongly agree)	5.9 (1.1)	6.2 (0.8)
	I plan to ask for a cancer family history (strongly disagree to strongly agree)	5.3 (0.8)	6.3 (0.6)

^a^All items were answered on a 7-point scale with a higher score indicating a more positive attitude toward the described behavior.

^b^SD: standard deviation.

^c^Item has been rescored from a 5-point to a 7-point scale.

**Table 5:** Attitude, beliefs, and intentions toward investigating a cancer family history (change scores).

Scale and item^a^		Change^b^	*z*	*P* value
**Beliefs about capabilities**				
	I have the ability to ask for a cancer family history (strongly disagree to strongly agree)	Decrease: 15 Increase: 14 Ties: 46	−0.19	.85
	I am confident that I could ask for a cancer family history (strongly disagree to strongly agree)	Decrease: 17 Increase: 15 Ties: 43	−0.10	.92
	For me, asking for a cancer family history would be (extremely difficult to extremely easy)^c^	Decrease: 24 Increase: 5 Ties: 46	−2.90	.004
**Social influences**				
	To the best of my knowledge, the proportion of colleagues who will ask for a cancer family history would be (0%-20%/ or 20%-40% or 40%-60% or 60%-80% or 80%-100%)^d^	Decrease: 5 Increase: 16 Ties: 54	−2.62	.009
	Now think about a coworker who you respect as a professional. In your opinion, does he or she ask for a cancer family history (never to always)	Decrease: 13 Increase: 14 Ties: 48	−0.37	.72
	Most persons who are important for me in the profession would ask for a cancer family history (strongly disagree to strongly agree)	Decrease: 5 Increase: 25 Ties: 45	−3.71	.001
**Beliefs about consequences**				
	Overall, I think that asking for a cancer family history from a medical point of view is (useless to useful)	Decrease: 13 Increase: 27 Ties: 35	−2.51	.01
**Moral norm**				
	Asking for a cancer family history is the right thing to do from a medical perspective (strongly disagree to strongly agree)	Decrease: 11 Increase: 26 Ties: 37	−2.73	.006
**Intention**				
	I intend to ask for a cancer family history (strongly disagree to strongly agree)	Decrease: 15 Increase: 27 Ties: 33	−2.71	.007
	I plan to ask for a cancer family history (strongly disagree to strongly agree)	Decrease: 2 Increase: 55 Ties: 18	−6.60	.001

^a^All items were answered on a 7-point scale with a higher score indicating a more positive attitude towards the described behavior.

^b^number of individuals that decreased or increased or stayed the same from T0 to T1, n=74.

^c^in the other direction: significant decrease in attitude.

^d^Item has been rescored from a 5-point to a 7-point scale.

**Table 6:** Participants’ expectations regarding the e-learning module.

Item^a^	T0 (n=123) Mean (SD^b^)	T1^c^ (n=93) Mean (SD)
I expect that the content of this e-learning is usable in clinical practice	5.3 (1.2)	5.7 (1.0)
I expect that the benefits of participating in this education via the Internet outweigh the disadvantages	5.4 (1.1)	5.7 (1.0)
I expect that participation in this education via the Internet will offer me the opportunity to organize my work more effectively	5.2 (1.1)	5.6 (1.1)

^a^7-point scale: strongly disagree (1) to strongly agree (7).

^b^SD: standard deviation.

^c^At T1, participants were asked if these expectations were fulfilled. For example, “I expected that participating in this education via the Internet would allow me to spend more time on other activities.”

#### Perceived Timing and Time Constraints

Out of all participants, 64% thought that the e-learning came at the right point in time, whereas 34% thought it came too late, and 2% thought it came too early in their educational track. In addition, 51% participants took 15-30 min to complete the e-learning, 43% took 30-45 min, 5% took 45-60 min, and 1% took 0-15 min.

Furthermore, 86% participants reported that the length of the e-learning module was exactly right, whereas 9% thought it was too lengthy, and 5% thought it was too short.

#### Design, Technical Usability, and Technical Problems

Participants completed the e-learning module on a computer or laptop (83%), mobile phone (14%), or tablet computers (3%). The majority of participants (85%) completed the e-learning module in 1 session, whereas 15% took two or more turns to finish it.

On a 5-point scale, participants evaluated the e-learning module as well-developed (mean 3.9 [SD 0.6]), user-friendly (mean 4.1 [SD 0.6]), nice (mean 3.7 [SD 0.7]), readable (mean 3.9 [SD 0.6]), and usable (mean 4.0 [SD 0.6]). On a 7-point scale, participants evaluated the language in the e-learning module as understandable (mean 5.8 [SD 0.8]) and the instructions in the e-learning as understandable (mean 5.8 [SD 0.7]), clear (mean 5.8 [SD 0.8]), useful (mean 5.7 [SD 0.7]), and complete (mean 5.7 [SD 0.8]).

Out of all surgeons, 21% reported having encountered technical problems, which could be categorized as related to readability, display on the mobile phone, loading of pages, and the “game” element (see below) not working properly. On the basis of this information, we adapted the game before inviting the gastroenterologists; none of the participants in this latter group experienced any technical problems.

#### Evaluation of Clinical Usability and Content

Table 6 shows the participants’ expectations regarding the e-learning module at T0 and, if these were fulfilled, at T1. At T0, participants had high expectations (mean 5.2-5.4 on a scale from 1-7) regarding the usability of the content, the benefits of education via Internet, and organizing their work more effectively. At T1, these expectations were fulfilled (mean 5.6-5.7 on scale 1-7) for all 3 items.

Case examples were evaluated as follows: clear 5.5 (SD 0.9), helpful 5.3 (SD 0.8), complete 5.2 (SD 1.1), and realistic 5.3 (SD 1.0) (7-point Likert scale, not at all to very much).

According to the surgeons, the most useful elements of the e-learning module were *short cases* (54%; ie, a case description in which the participant needs to judge if the patient needs to be referred for genetic counseling) and the *overview of helpful devices or aids* (45%; ie, links to relevant information in apps, checklists, and questionnaires with reflective questions). Least useful were the *game on misunderstandings* (70%; ie, a description of the most common misunderstandings that patients have about genetic testing, based on clicking on rolling balls within a certain time frame), and the *barriers* (56%; ie, participants could click on words in a word cloud presenting the most common barriers and how to overcome these). On the basis of this information, we changed the “misunderstandings” in the game component, that is, in the revised version; pictures of “patients” were shown with a text balloon reflecting their misunderstandings. Additionally, explanations about the misunderstandings and how to deal with them were provided. Furthermore, the word cloud with the “barriers” was made voluntary instead of being obligatory (Table 2).

According to gastroenterologists, the most useful elements of the e-learning module were the *short cases* (40%), *the overview of criteria for referral* (40%), *the long cases* (40%; ie, two clinical scenarios in the form of a comic with questions), and *the overview of helpful devices or aids* (30%). Least useful to gastroenterologists were the *barriers* (50%), the *pretest* (40%), the *misunderstanding cases* (30%), and the *misunderstanding pictures* (30%; ie, pictures of patients expressing misunderstandings).

Out of the surgeons, 21% indicated that they “missed” something: this was mainly categorized as more background information (eg, more in-depth information on the background of hereditary problems), more overviews (eg, overview of the Lynch criteria), or more information about referral (eg, more insight into when a referral is needed). On the basis of this information, an overview of helpful resources (eg, guidelines and assessment tools for cancer family history) was added. None of the gastroenterologists indicated that they missed something.

## Discussion

### Principal Findings

This study investigated the feasibility of an interactive problem-based e-learning module for gastroenterologists and surgeons (in training) aimed at improving *knowledge* of hereditary CRC, *attitude* toward, and *comprehension of communication skills* needed for a cancer family history assessment. The study provides relevant insights for researchers and teachers in the field of online learning. A unique innovative e-learning has been developed, using problem-based comics displaying realistic doctor-patient conversations, which activate participants to elaborate on what would be appropriate behavior.

The e-learning led to the intended improvements in attitude toward assessment of cancer family history, knowledge on CRC criteria for referral to genetic counseling, and comprehension of communication skills. *Attitude* toward a cancer family history assessment became more positive on 6 out of the 10 items. *Knowledge* on hereditary cancer and *comprehension* of communication skills showed significant improvement. Studies aimed at health professionals investigating the effect of e-learning modules on attitude and knowledge showed similar effects [14,22-26] in, for example, end-of-life care, reducing antibiotic prescribing, error reporting, behavior change psychology, and addressing unhealthy alcohol use. Studies investigating the effect of e-learning on communication skills or comprehension of skills are scarce. However, McCarthy et al showed that their program resulted in an improvement of skills in knowing when and how to complete incident forms and disclosing errors [14]. Daetwyler et al showed that their module improved the skill of breaking “bad news” in a setting with a simulated patient [15].

Participants in this study self-reported that they learned more about accurately assessing cancer family history than about hereditary CRC. Importantly, asking for a cancer family history was perceived as significantly less easy for participants after following the e-learning module than before the training. Thus, although the comprehension of communication skills increased, the participants felt more insecure about asking for a cancer family history. A better or increased understanding of what is needed for an accurate cancer family history assessment may have influenced the confidence in their own skills. In other words, participants may have become aware of the gaps in their skills (ie, consciously incompetent); a positive consequence of this may be an increased willingness to change behavior and learn from future experiences [27].

The evaluation of our e-learning module shows that the e-learning was well received and that participants were positive about both the design and content. The positive evaluation of the e-learning may be further increased by adding more problem-based cases to increase the variety of cases and to stimulate recall of the referral criteria. Most surgeons and gastroenterologists found the e-learning module attractive and useful, and would recommend it to others. However, the majority indicates that they are not willing to pay for the e-learning module, which may complicate the implementation of e-learning in continuing medical education. Therefore, it is important to incorporate the e-learning module in existing educational tracks. Moreover, as medical students and nurses may benefit from this e-learning, the module may be incorporated in their educational tracks. Furthermore, a blended learning (ie, adding a traditional communication training session to the e-learning module) may increase the application of the learned skills into clinical practice and incorporate the learned skills cognitively.

A limitation of this study is the restricted dataset, as some participants did not fill in the postquestionnaire. This may be because of lack of time or technical reasons, that is, not being able to complete the e-learning module. Therefore, the number of participants reporting technical problems may be lower than those actually experiencing technical problems. In addition, we did not test the actual impact of the e-learning module on communication skills (eg, using a simulated patient). Potentially, this e-learning should be integrated in a blended learning model to enable a greater effect on participant’s skills; in this way, participants can practice with the learned skills and thereby transfer their skills to daily practice.

A strength of the study is that we were able to demonstrate that (at least the basic) communication skills have the potential to be trained via an e-learning module. Another limitation is that the same test was used to assess knowledge before and after completing the e-learning module, which may have caused testing bias. However, as we did not provide feedback about the first (T0) test and the second test was (on average) about 30 min later, this effect is probably small. On the other hand, this short time frame may have led the test to function as a memory test rather than a test of their capacities. A test after a few weeks, following the e-learning module, would have given more insight in the learning effect.

Our problem-based e-learning on recognizing hereditary predisposition in patients with CRC was feasible for surgeons-in-training and gastroenterologists. The e-learning module is now ready for use and appears to be a useful tool to improve knowledge on hereditary CRC and attitudes toward and comprehension of a cancer family history assessment.

Future studies should evaluate whether the e-learning module has a beneficial effect on more adequate referral of at-risk patients to genetic counseling, resulting in more preventive screening, better prevention, and or timely diagnosis in patients and their family members. The results of this study are promising and warrant additional research on how communication skills can be further addressed in online learning.

## Appendix

Multimedia Appendix 1 Screenshots of a comic with questions.

## Abbreviations

CRC: colorectal cancer
SD: standard deviation
