# Supplementary material for: An E-Learning Module to Improve Nongenetic Health Professionals’ Assessment of Colorectal Cancer Genetic Risk: Feasibility Study
Source: JMIR Med Educ. 2017 Dec 18;3(2):e24. doi: 10.2196/mededu.7173 (PMC5748476; doi:10.2196/mededu.7173)
Supplement: Multimedia Appendix 1 [file mededu_v3i2e24_app1.pdf]

Multimedia appendix 1: Screenshots of a comic with questions

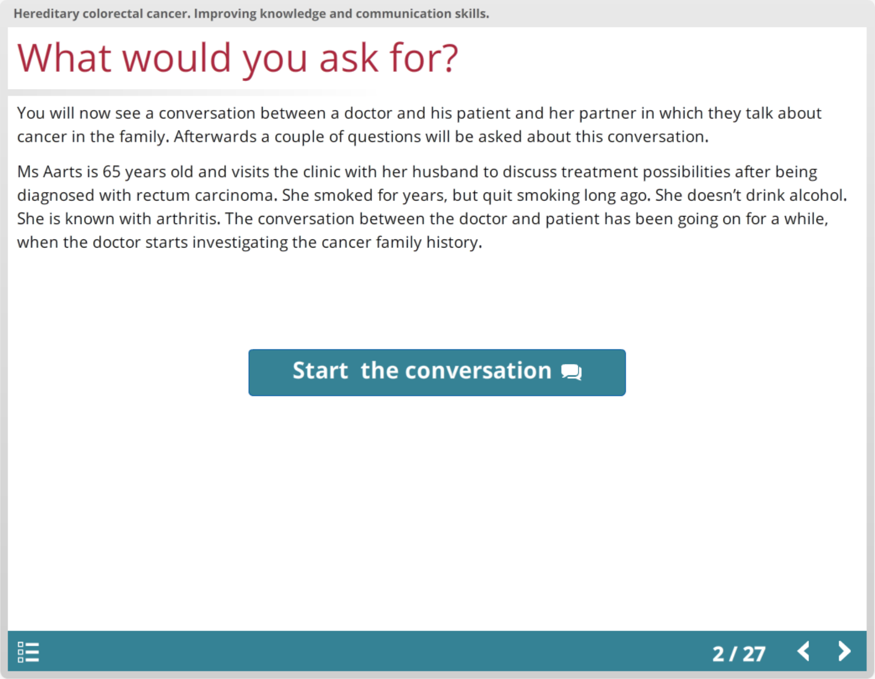

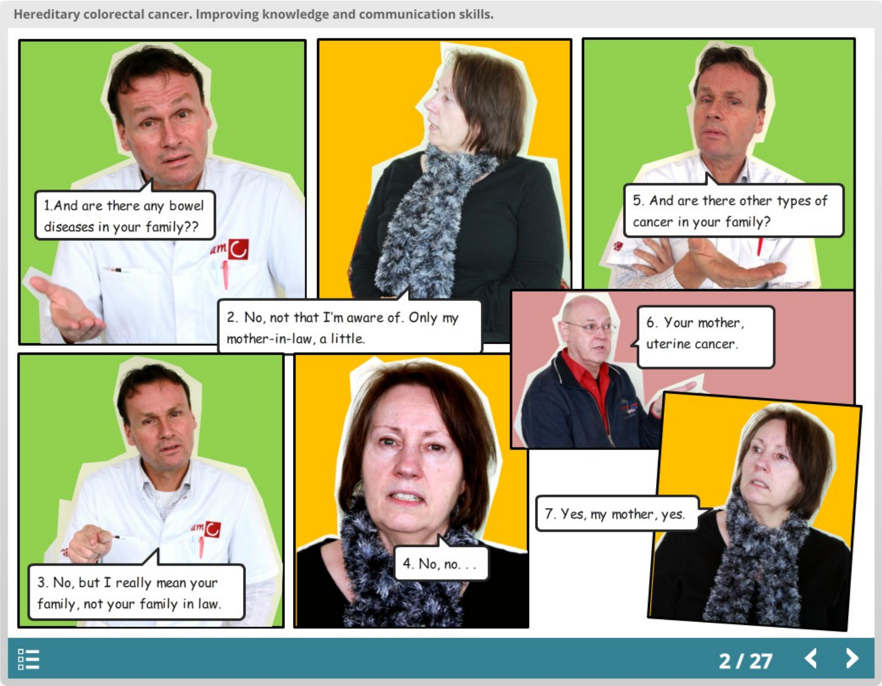

Do you have enough information?

Do you have enough information to determine if the patient should be referred for genetic counseling?

- ○ Yes, I have enough information. The patient should not be referred.
- ○ No, I don't have enough information
- ◉ Yes, I have enough information. The patient should be referred.

Check answer

Hereditary colorectal cancer. Improving knowledge and communication skills.

Do you have enough information?

Do you have enough information to determine if the patient should be referred for genetic counseling?

- ○ Yes, I have enough information. The patient should not be referred.
- ○ No, I don't have enough information
- ◉ Yes, I have enough information. The patient should be referred.

Sorry

Sorry, you do not have enough information yet to determine if a referral is necessary. Based on this information you cannot support sufficiently why this patient should be referred or not.

Continue

3 / 27

Get a clear picture

Which question or questions could you ask now to get a clearer picture about the cancer in the family of this patient?

- [x] 1. Did your mother have metastases of the uterine cancer?
- [ ] 2. Did your mother have cervical cancer, as often is discovered with a smear through the national screening program, or uterine cancer?
- [x] 3. How old was your mother when she died?
- [x] 4. At which age did your mother get cancer?

Check answer

Hereditary colorectal cancer. Improving knowledge and communication skills.

Get a clear picture

Which question or questions could you ask now to get a clearer picture about the cancer in the family of this patient?

- [x] 1. Did your mother have metastases of the uterine cancer?
- [ ] 2. Did your mother have cervical cancer, as often is discovered with a smear through the national screening program, or uterine cancer?
- [x] 3. How old was your mother when she died?
- [x] 4. At which age did your mother get cancer?

Wrong. The right answers are 2 and 4. Option 3 is not completely wrong, but option 4 is better. Option 1 is wrong.

There are several possibilities. First of all, it is important to get more clarity about the likelihood that it is indeed endometrial cancer. Many patients confuse endometrial cancer with a cervix carcinoma (which is often discover with a smear, through screening) or with ovarium cancer. Endometrial cancer is a Lynch-associated tumor while a cervix carcinoma is not.

When the mother of the patient indeed had endometrial cancer it is important to know at which age she got it. This will determine if a referral is necessary. The age at which the mother died will give you an impression as well, but there could have been years between diagnosis and death.

Metastases are irrelevant for referral for genetic counseling.

Continue

4 / 27
